# Preferential desulfurization of dibenzyl sulfide by an isolated *Gordonia* sp. IITR100

**DOI:** 10.1007/s13205-014-0221-1

**Published:** 2014-05-14

**Authors:** Abrar Ahmad, Ashok Kumar Chauhan, Hari Narayan Kushwaha, Saleem Javed, Ashwani Kumar

**Affiliations:** 1 Environmental Biotechnology Division, CSIR-Indian Institute of Toxicology Research, M.G. Marg, Lucknow, 226001 India; 2 Pharmacokinetics and Metabolism Division, Central Drug Research Institute, Lucknow, 226001 India; 3 Department of Biochemistry, Faculty of Science, Jamia Hamdard, New Delhi, 110062 India

**Keywords:** Biodesulfurization, Dibenzyl sulfide, *Gordonia* sp. IITR100, *dsz*ABC

## Abstract

Several organosulfur compounds are present in the crude oil, and are required to be removed before its processing into transport fuel. For this reason, biodesulfurization of thiophenic compounds has been studied extensively. However, studies on the sulfide compounds are scarce. In this paper, we describe desulfurization of a model sulfidic compound, dibenzyl sulfide (DBS) by an isolated *Gordonia* sp. IITR100. The reaction was accompanied with the formation of metabolites dibenzyl sulfoxide, dibenzyl sulfone and benzoic acid. Studies with recombinant *E. coli* revealed that enzyme DszC of this isolate metabolizes DBS into dibenzyl sulfoxide and dibenzyl sulfone, but the reaction downstream to it is mediated by some enzyme other than its DszA. In reactions where DBS and dibenzothiophene (DBT) were present together, both IITR100 and recombinant *E. coli* exhibited preference for the desulfurization of DBS over DBT. The newly identified capability of IITR100 for desulfurization of both thiophenic and sulfidic compounds suggests its potential use in improved desulfurization of petroleum fractions.

## Introduction

Several organosulfur compounds that include thiols, mercaptans, thiophenes and sulfides are present in crude petroleum (Peinder et al. 2010; Liu et al. 2010; Lam et al. 2012) which are required to be removed, prior to its use in automobiles and industry. For this reason, biodesulfurization of crude oil and its fractions has been studied extensively (Van Hamme et al. 2003a; Gupta et al. 2005; Kilbane 2006; Mohebali and Ball 2008; Xu et al. 2009). Most of these have been done on model thiophenic compound dibenzothiophene (DBT), and details of the desulfurization pathway, responsible enzymes and their genes have been reviewed (Denome et al. 1994; Piddington et al. 1995). Briefly, desulfurization of DBT is mediated by a ‘4S’ pathway that includes serial activity of enzymes DszC, DszA and DszB, leading to the formation of hydroxy-biphenyl as end product (Van Hamme et al. 2003a; Gupta et al. 2005; Mohebali and Ball 2008; Xu et al. 2009).

Studies on the desulfurization of sulfide compounds, however, are scarce (Van Hamme et al. 2003b, 2004; Kirkwood et al. 2005). The possible reason could be that the archetypal *Rhodococcus* sp. IGTS8, characterized extensively for the desulfurization of DBT (Mohebali and Ball 2008; Xu et al. 2009), was not found to be able to mediate the desulfurization of the model compound dibenzyl sulfide—DBS (Van Hamme et al. 2004). In contrast, *Rhodococcus* sp. strain JVH1 that was isolated for its ability for desulfurization of DBS does not utilize DBT as a sulfur source (Van Hamme et al. 2004). Consistent with this finding, genome sequence of JVH1 does not contain any genes that are similar to *dsz*ABC (Brooks and Van Hamme 2012). In this report, we describe the desulfurization of both DBT and DBS by an isolated strain *Gordonia* sp. IITR100.

## Materials and methods

### Chemicals

Dibenzothiophene (DBT), dibenzothiophene sulfone (DBTOO), dibenzyl sulfide (DBS), dibenzyl sulfone (DBSOO) and acetonitrile were purchased from Sigma-Aldrich (St. Louis, MO, USA). Dibenzyl sulfoxide was from MERCK-Schuchardt (Mumbai, India). All the other chemicals were of analytical grade.

### Bacterium

A bacterium *Gordonia* sp. IITR100 (16S ribosomal RNA accession no; GU084407), obtained earlier by selective enrichment on dimethyl DBT from an oil-contaminated soil that was present around a refinery in Gujarat, India (Singh et al. 2011), was used. Nucleotide sequence of its *dsz*ABC genes (Accession no; KC693733.1) is >99 % identical to the corresponding genes of *Gordonia alkanivorans* strains 1B (Alves et al. 2007) and RIPI90.

### Desulfurization by IITR100

For DBS desulfurization, 20 flasks, each containing 20 ml medium-1 (Na_2_HPO_4_, 2.0 g; KH_2_PO_4_, 1 g; MgCl_2_·6H_2_O, 0.4 g; (NH_4_)_2_C_2_O_4_, 4.25 g; Al(OH)_3_, 0.1 g; SnCl_2_·2H_2_O, 0.5 g; KI, 0.05 g; LiCl, 0.01 g; MnCl_2_·4H_2_O, 0.8 g; H_3_BO_3_, 0.05 g; ZnCl_2_, 0.1 g; CoCl_2_·6H_2_O, 0.1 g; NiCl_2_·6H_2_O, 0.1 g; BaCl_2_, 0.05 g; (NH_4_)_6_Mo_7_O_24_·4H_2_O, 0.05 g, 17.1 g Sucrose, per liter), along with 0.3 mM of DBS as sulfur source, were inoculated with IITR100. Three flasks for each time point, i.e., after 0, 2, 4, 6, and 8 days of incubation were removed. After estimation of growth (OD_600_), the reaction was stopped by acidification to pH < 2.0, and levels of residual DBS and the formed metabolites were analyzed. Un-inoculated flasks were run in parallel and were processed likewise.

Similar experiment was set up for studying the desulfurization of DBT, except that 0.3 mM DBT was used as sulfur source. For experiments, where desulfurization of DBT and DBS was to be evaluated, when these were present together, similar set up was used except that 0.3 mM each of DBS and DBT was used as sulfur source.

### Desulfurization by recombinant *E. coli* cells

Recombinant *E. coli* DszA and *E. coli* DszC that were expressing DszA and DszC, respectively, were prepared by ligation of the amplified *dsz*A and *dsz*C genes (Table 1), respectively, with pET28a, and were followed by their cloning in *E. coli* BL21 as described earlier (Singh et al. 2007). After induction by IPTG, the cells from one liter culture were harvested by centrifugation at 3,500*g*, washed with 50 ml medium-1 and suspended in 100 ml of the same medium. For evaluation of the activity of DszC with the substrates, 0.3 mM of DBT, DBS or mixture of DBT and DBS were incubated with 10 ml suspension of the *E. coli* DszC. After incubation at 30 °C for different time periods, the reaction was stopped by acidification and residual substrate along with the formed metabolites was analyzed, as described below. Metabolism of DBTOO and DBSOO with *E. coli* DszA was also evaluated likewise.

**Table 1:** List of primers used in the present study

No.	Name	Sequence	Properties
1	*dsz*AF	GGAATTCCATATGGCTCAACGGCGACAACTGCATCTGGCCGGTTTC	Contains 1-36 bases of 5′end of *dsz*A and is preceded by site for *Nde*I
2	*dsz*AR	CCGCTCGAGGTGTGTCGAGGATGCCGGTATCAAGTTCTGAACCGG	Contains 1-33 bases of 3′end of *dsz*A and is preceded by site for *Xho*I
3	*dsz*CF	GGAATTCCATATGACTCTGTCCGTTGAAAAGCAGCACGTTCG	Contains 1-32 bases of 5′end of *dsz*C and is preceded by site for *Nde*I
4	*dsz*CR	CCGCCCAAGCTTCTAGGAGGTGAAGCCGGGAATCGGGTA	Contains 1-27 bases of 3′end of *dsz*C and is preceded by site for *Hind*III

Sequences were based on those of *dsz*ABC genes of IITR100 (Accession No.-KC693733.1)

### Analytical methods

The acidified culture medium was extracted three times with equal volume of ethyl acetate. The residue obtained after complete evaporation of the solvent was dissolved in acetonitrile and a suitable aliquot was used for HPLC analysis, carried on a Waters instrument (Milford, MA, USA), equipped with PDA 996 detector and LiChrospher^®^ 100 RP-18 columns (5 µm, 4.6 × 250 mm). It was run at 28 °C, using 100 % acetonitrile as mobile phase at the flow rate 0.50 ml/min.

## Results

### Desulfurization of dibenzyl sulfide by IITR100

**Fig. 1:**
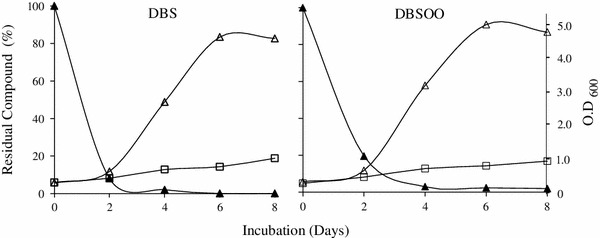
Desulfurization of dibenzyl sulfide (DBS) and dibenzyl sulfone (DBSOO), in the presence of IITR100. Residual compound (*closed triangles*), and growth of IITR100 in presence of DBS/DBSOO (*open triangles*) or their absence (*open squares*) are also shown

**Fig. 2:**
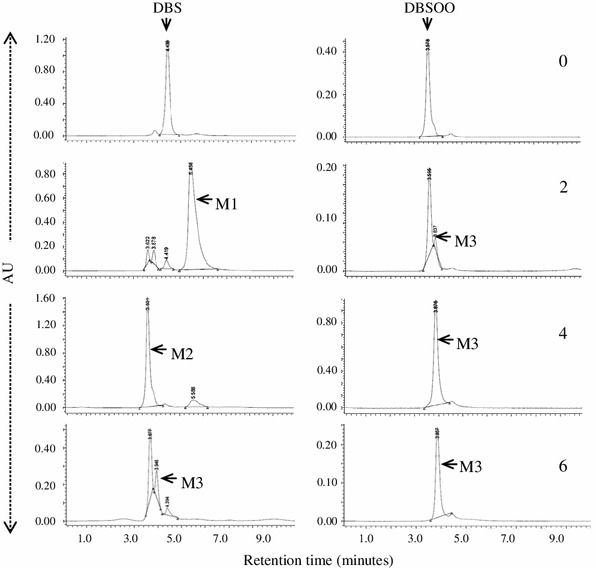
Metabolism of dibenzyl sulfide (DBS) and dibenzyl sulfone (DBSOO) in the presence of IITR100, after 0, 2, 4 and 6 days of incubation. Retention times 5.62, 3.63 and 3.94 min of the formed metabolites M1, M2 and M3, respectively, were same as for dibenzyl sulfoxide (DBSO), dibenzyl sulfone (DBSOO) and benzoic acid

**Fig. 3:**
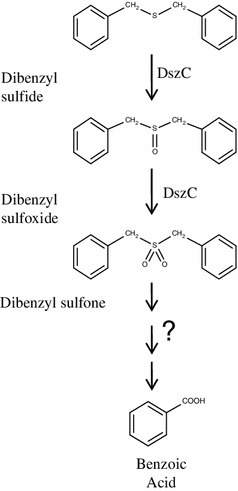
Proposed pathway for desulfurization of DBS by IITR 100

The strain IITR100 was able to grow in liquid medium using either DBS or DBS sulfone as source of sulfur, whose levels declined progressively with increasing time periods (Fig. 1). The growth curves on both of these compounds were comparable, i.e., there was a lag period of 2 days and maximal growth (5.0 OD_600_) was seen after 6 days of incubation. The growth with DBS was accompanied with the formation of three metabolites, i.e., M1, M2 and M3, which were subsequently identified as DBS sulfoxide, DBS sulfone, and benzoic acid, respectively, based on their co-migration on HPLC with authentic standards. Identity of M3 was further confirmed by its mass spectrum, which matched well with that of the authentic standard (data not shown). Similar to DBS, growth of IITR100 cells on DBS sulfone was accompanied with the formation of metabolite M3. Level of the formed M3 was highest after 4 days of incubation with IITR100, but decreased thereafter (Fig. 2), suggesting it to be an intermediary metabolite and not the end product. Based on the metabolites formed, desulfurization of DBS to benzoic acid appears to proceed via the formation of DBS sulfoxide and DBS sulfone (Fig. 3).

### Transformation of DBT and DBS by recombinant *E. coli*

**Fig. 4:**
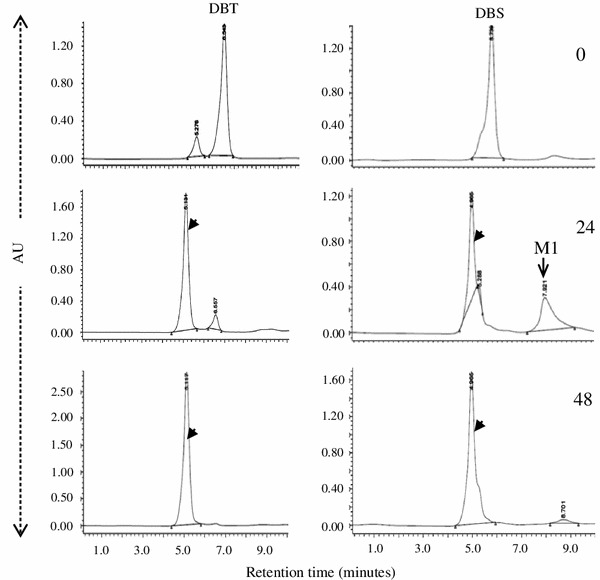
Metabolism of DBT and DBS by recombinant *E. coli* DszC to their corresponding sulfones (shown by *arrowheads*), after 0, 24 and 48 h of incubation. Metabolite M1 is the intermediary dibenzyl sulfoxide

**Fig. 5:**
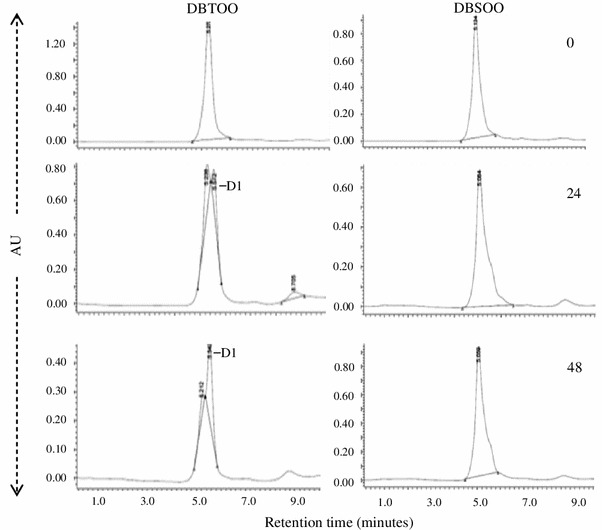
Metabolism of DBT sulfone (DBTOO) and DBS sulfone (DBSOO) by *E. coli* DszA. Formation of a metabolite D1 after 24 and 48 h incubation with DBTOO, but no metabolism of DBSOO under the same conditions, is also shown

Incubation with the recombinant *E. coli* DszC that was harboring *dsz*C gene of IITR100 led to the oxidation of DBT and DBS to the corresponding sulfones (Fig. 4). A transient accumulation of DBS sulfoxides was observed after 24 h incubation, but was metabolized by 48 h. Incubation of DBT sulfone with recombinant *E. coli* DszA that was harboring *dsz*A gene of IITR100 led to its transformation into a metabolite DI (Fig. 5), possibly DBT sulfinite, but their incubation under the same conditions did not cause any metabolism of DBS sulfone.

### Metabolism of DBT and DBS, when present alone or together

**Fig. 6:**
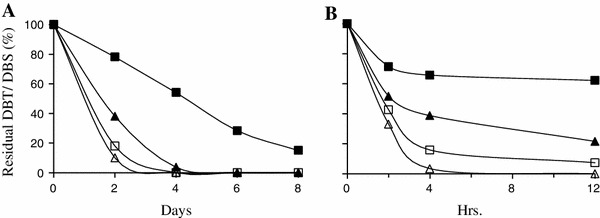
Desulfurization/metabolism of DBS (*triangles*) and DBT (*squares*) with IITR100 (Panel **a**) and with recombinant *E. coli* DszC (Panel **b**), when present separately (*open*) or together (*closed*)

When the two chemicals were evaluated separately, desulfurization of DBS by IITR100 was marginally better than DBT, and both the chemicals were utilized completely by 3 and 4 days of incubation, respectively (Fig. 6a). But, when the two chemicals were present together, an enhanced preference for desulfurization of DBS was observed. Here, while nearly all of DBS was metabolized after 4 days of incubation, ~50 % of DBT was still present in the medium after the same period. Similarly, preferential metabolism of DBS was also observed when the metabolism of DBT and DBS by *E. coli* DszC cells was evaluated. Thus, when the two chemicals were present individually, ~nil and 10 % of DBS and DBT, respectively, remained in the reaction medium after 12 h of incubation (Fig. 6b). But when the two chemicals were present together, the difference in their metabolism became sharper and ~30 and 70 % of DBS and DBT remained after the same time period.

## Discussion

The study was designed to study the desulfurization of a model sulfide compound dibenzyl sulfide (DBS) by an isolated strain *Gordonia* sp. IITR100, when it is present alone or in the presence of a thiophenic compound DBT. Results revealed that the desulfurization of DBS proceeds via the formation of metabolites DBS sulfoxide, DBS sulfone and benzoic acid. While the conversion of DBS to DBS sulfone is mediated by the activity of DszC, further metabolism of the formed DBS sulfone is mediated by some enzyme other than DszA. The results are in agreement with earlier studies, where formation of the same metabolites has been reported by the activity of *Rhodococcus* sp. strains JVH-1(Van Hamme et al. 2004) and K1bD (Kirkwood et al. 2005). The enzymes responsible for the activity, however, were not studied in these bacteria. Metabolism of DBS to DBS sulfone by DszC that was obtained from the strain IGTS8 has been shown earlier (Lei and Tu 1996), but the reaction was extremely slow and only 6 % of DBS was converted into DBS sulfone by the end of the reaction. Nearly 70 % of it remained as the intermediary metabolite DBS sulfoxide. In contrast, the metabolism of DBS to DBSOO by *E. coli* DszC in the present study was rapid and no significant accumulation of DBS sulfoxide was observed at the end of the reaction (Fig. 4).

Based on the analogy with DBT desulfurization, it has been proposed that the formed DBS sulfone might undergo further metabolism by enzyme DszA (Van Hamme et al. 2004). Results of the present study, however, revealed that although IITR100 is able to cause desulfurization of DBS, its DszA has no activity towards the metabolism of DBS sulfone. It suggests that this reaction in IITR100 might proceed by some enzyme that is distinct from DszABC. The same might also be true for the previously characterized DBS-metabolizing strain *Rhodococcus* sp. JVH1, which does not carry *dsz*ABC genes (Brooks and Van Hamme 2012).

Interestingly, although IITR100 was isolated initially for its activity of DBT desulfurization, it exhibits preference for the desulfurization of DBS over DBT, possibly due to the similar preference by its DszC (Fig. 6). Similarly, a strain *Paenibacillus* sp. A11-2, isolated by enrichment on DBT, exhibits 2-threefold higher activity towards benzothiophene (Konishi et al. 2000). Thus, it is a possibility that several desulfurizing strains described in the literature, whose *dszABC* genes are different from the archetypal *Rhodococcus* IGTS 8, might actually have preference for other organosulfur compounds, which can be evaluated using their binary or bigger combination of the substrates.

## Conclusion

- *Gordonia* sp. strain IITR100 mediates desulfurization of dibenzyl sulfide (DBS).
- Metabolism of DBS to DBS sulfone is mediated by enzyme DszC.
- Further metabolism of DBS sulfone takes place with some enzyme other than DszA.
- IITR100 as well as recombinant *E*. *coli* DszC exhibits preference for the desulfurization of DBS over DBT, when present together.
- IITR 100 has potential for desulfurizing both aliphatic and aromatic organosulfur compounds.
- This strain could be used for biodesulfurization of broad substrate range of organosulfur compounds in petroleum fractions.
